# Supplementary material for: Limitation of amino acid availability by bacterial populations during enhanced colitis in IBD mouse model
Source: mSystems. 2023 Nov 1;8(6):e00703-23. doi: 10.1128/msystems.00703-23 (PMC10746178; doi:10.1128/msystems.00703-23)
Supplement: Figure S1 — Clustered heatmap of all mice with MAG detection ratios. [file msystems.00703-23-s0001.pdf]

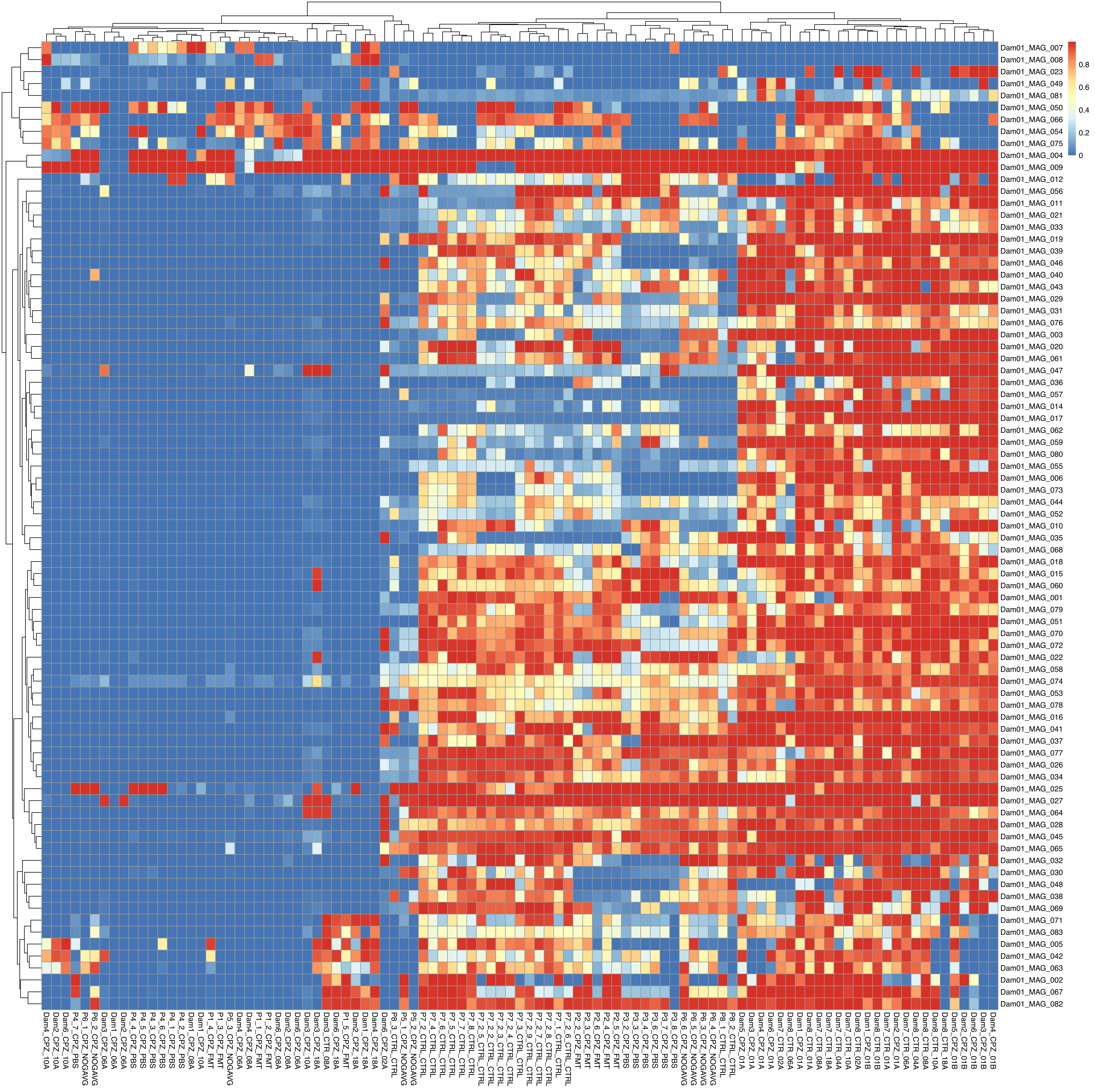

Supplementary Figure S1. Clustered heatmap of IL-10 KO with the non-redundant MAGs of all pups and corresponding dams, before and after antibiotic treatments. Color scale indicates the level of detection of each MAG corresponding to each sample, the x-axis are all non-redundant MAGs and the y-axis are individual mouse samples. Samples labeled Dam correspond to only Dam samples either before (B) or after (A) antibiotic exposure. Pup samples (P) are labeled by the treatment group and all samples were taken at week 23 of age. Overall, there is a large shift in MAG detections seen in Dam samples after antibiotic exposure, and clusters tightly with dysbiotic pup groups (No Gavage and PBS Gavage). Also note that the FMT pups closely align with Control pups also seen in Figure 4.
