# Supplementary material for: Limitation of amino acid availability by bacterial populations during enhanced colitis in IBD mouse model
Source: mSystems. 2023 Nov 1;8(6):e00703-23. doi: 10.1128/msystems.00703-23 (PMC10746178; doi:10.1128/msystems.00703-23)
Supplement: Figure S2 — Gene ontology comparisons of each mouse treatment group. [file msystems.00703-23-s0002.pdf]

Supplementary Figure S2. Rank-based Gene Ontology Analysis with Adaptive Clustering of control and FMT groups.

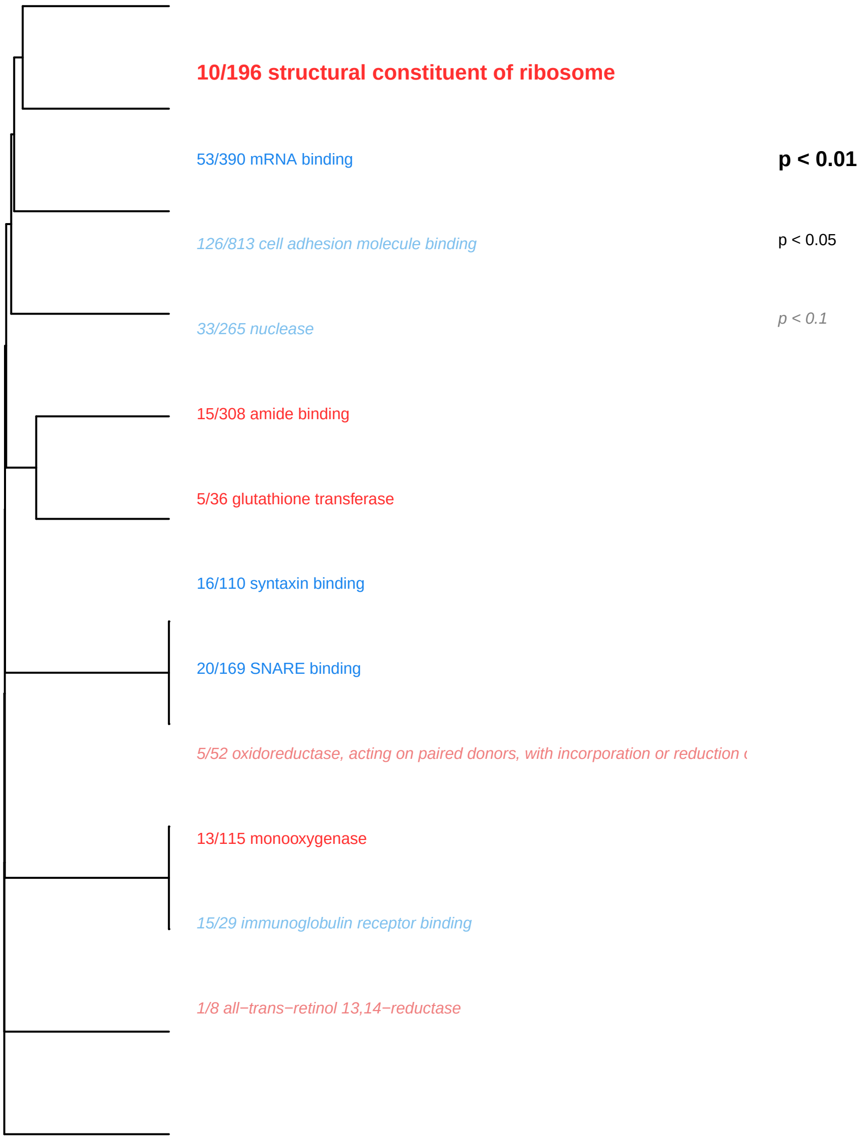

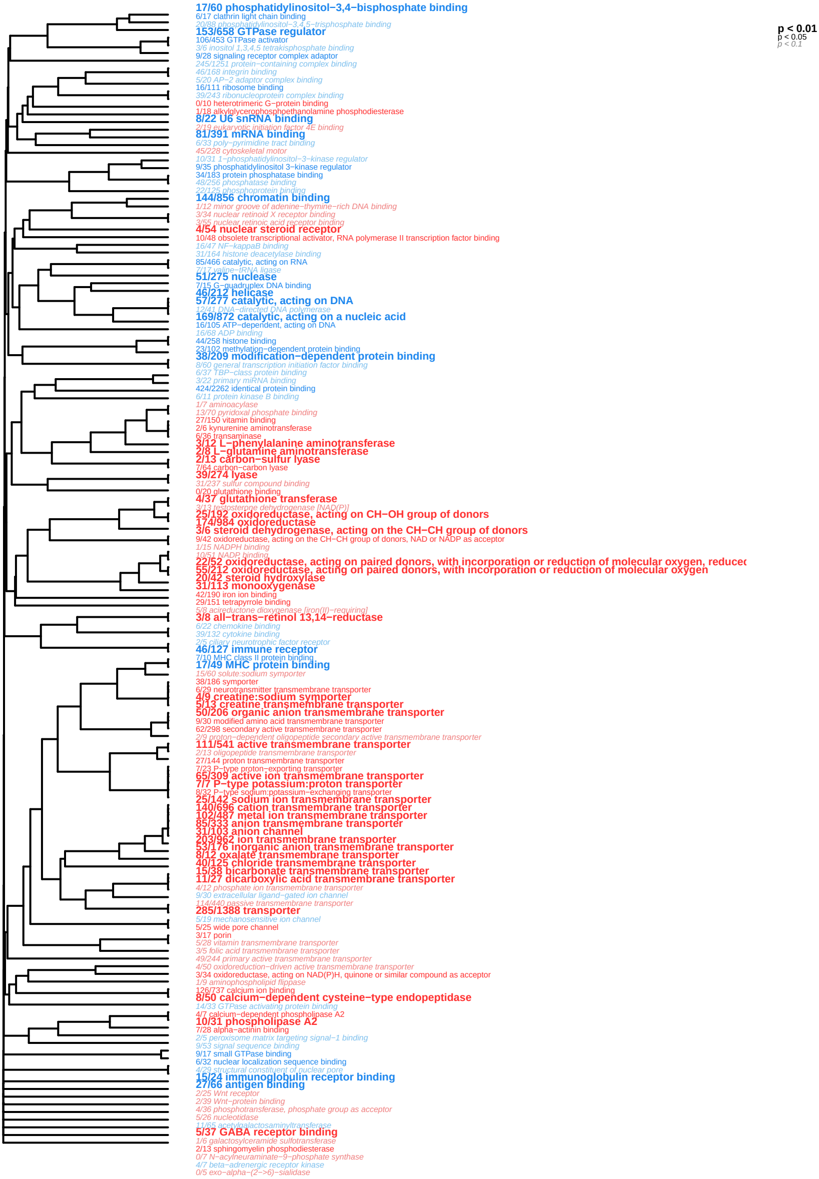
p < 0.01
p < 0.05
p < 0.1
17/60 phosphatidylinositol−3,4−bisphosphate binding
6/17 clathrin light chain binding
20/88 phosphatidylinositol−3,4,5−trisphosphate binding
153/658 GTPase regulator
106/453 GTPase activator
3/6 inositol 1,3,4,5 tetrakisphosphate binding
9/28 signaling receptor complex adaptor
245/1251 protein−containing complex binding
46/168 integrin binding
5/20 AP−2 adaptor complex binding
16/111 ribosome binding
39/243 ribonucleoprotein complex binding
0/10 heterotrimeric G−protein binding
1/18 alkylglycerophosphoethanolamine phosphodiesterase
8/22 U6 snRNA binding
2/19 eukaryotic initiation factor 4E binding
81/391 mRNA binding
6/33 poly−pyrimidine tract binding
45/228 cytoskeletal motor
10/31 1−phosphatidylinositol−3−kinase regulator
9/35 phosphatidylinositol 3−kinase regulator
34/183 protein phosphatase binding
48/256 phosphatase binding
22/125 phosphoprotein binding
144/856 chromatin binding
1/12 minor groove of adenine−thymine−rich DNA binding
3/34 nuclear retinoid X receptor binding
3/55 nuclear retinoic acid receptor binding
4/54 nuclear steroid receptor
10/48 obsolete transcriptional activator, RNA polymerase II transcription factor binding
16/47 NF−kappaB binding
31/164 histone deacetylase binding
85/466 catalytic, acting on RNA
7/17 valine−tRNA ligase
51/275 nuclease
7/15 G−quadruplex DNA binding
46/212 helicase
57/277 catalytic, acting on DNA
12/41 DNA−directed DNA polymerase
169/872 catalytic, acting on a nucleic acid
16/105 ATP−dependent, acting on DNA
16/68 ADP binding
44/258 histone binding
23/102 methylation−dependent protein binding
38/209 modification−dependent protein binding
8/60 general transcription initiation factor binding
6/37 TBP−class protein binding
3/22 primary miRNA binding
424/2262 identical protein binding
6/11 protein kinase B binding
1/7 aminoacylase
13/70 pyridoxal phosphate binding
27/150 vitamin binding
2/6 kynurenine aminotransferase
6/36 transaminase
3/12 L−phenylalanine aminotransferase
2/8 L−glutamine aminotransferase
2/13 carbon−sulfur lyase
7/64 carbon−carbon lyase
39/274 lyase
31/237 sulfur compound binding
0/20 glutathione binding
4/37 glutathione transferase
3/13 testosterone dehydrogenase [NAD(P)]
25/192 oxidoreductase, acting on CH−OH group of donors
174/984 oxidoreductase
3/6 steroid dehydrogenase, acting on the CH−CH group of donors
9/42 oxidoreductase, acting on the CH−CH group of donors, NAD or NADP as acceptor
1/15 NADPH binding
10/51 NADP binding
22/52 oxidoreductase, acting on paired donors, with incorporation or reduction of molecular oxygen, reducec
55/212 oxidoreductase, acting on paired donors, with incorporation or reduction of molecular oxygen
20/42 steroid hydroxylase
31/113 monooxygenase
42/190 iron ion binding
29/151 tetrapyrrole binding
5/8 acireductone dioxygenase [iron(II)−requiring]
3/8 all−trans−retinol 13,14−reductase
6/22 chemokine binding
39/132 cytokine binding
2/5 ciliary neurotrophic factor receptor
46/127 immune receptor
7/10 MHC class II protein binding
17/49 MHC protein binding
15/60 solute:sodium symporter
38/186 symporter
6/29 neurotransmitter transmembrane transporter
4/9 creatine:sodium symporter
5/13 creatine transmembrane transporter
50/206 organic anion transmembrane transporter
9/30 modified amino acid transmembrane transporter
62/298 secondary active transmembrane transporter
2/9 proton−dependent oligopeptide secondary active transmembrane transporter
111/541 active transmembrane transporter
2/13 oligopeptide transmembrane transporter
27/144 proton transmembrane transporter
7/23 P−type proton−exporting transporter
65/309 active ion transmembrane transporter
7/7 P−type potassium:proton transporter
8/32 P−type sodium:potassium−exchanging transporter
25/142 sodium ion transmembrane transporter
140/696 cation transmembrane transporter
102/487 metal ion transmembrane transporter
85/333 anion transmembrane transporter
31/103 anion channel
203/962 ion transmembrane transporter
53/176 inorganic anion transmembrane transporter
8/12 oxalate transmembrane transporter
40/125 chloride transmembrane transporter
15/38 bicarbonate transmembrane transporter
11/27 dicarboxylic acid transmembrane transporter
4/12 phosphate ion transmembrane transporter
9/30 extracellular ligand−gated ion channel
114/440 passive transmembrane transporter
285/1388 transporter
5/19 mechanosensitive ion channel
5/25 wide pore channel
3/17 porin
5/28 vitamin transmembrane transporter
3/5 folic acid transmembrane transporter
49/244 primary active transmembrane transporter
4/50 oxidoreduction−driven active transmembrane transporter
3/34 oxidoreductase, acting on NAD(P)H, quinone or similar compound as acceptor
1/9 aminophospholipid flippase
126/737 calcium ion binding
8/50 calcium−dependent cysteine−type endopeptidase
14/33 GTPase activating protein binding
4/7 calcium−dependent phospholipase A2
10/31 phospholipase A2
7/28 alpha−actinin binding
2/5 peroxisome matrix targeting signal−1 binding
9/53 signal sequence binding
9/17 small GTPase binding
6/32 nuclear localization sequence binding
4/29 structural constituent of nuclear pore
15/24 immunoglobulin receptor binding
27/66 antigen binding
2/25 Wnt receptor
2/39 Wnt−protein binding
4/36 phosphotransferase, phosphate group as acceptor
5/26 nucleotidase
11/65 acetylgalactosaminyltransferase
5/37 GABA receptor binding
1/6 galactosylceramide sulfotransferase
2/13 sphingomyelin phosphodiesterase
0/7 N−acylneuraminate−9−phosphate synthase
4/7 beta−adrenergic receptor kinase
0/5 exo−alpha−(2−>6)−sialidase

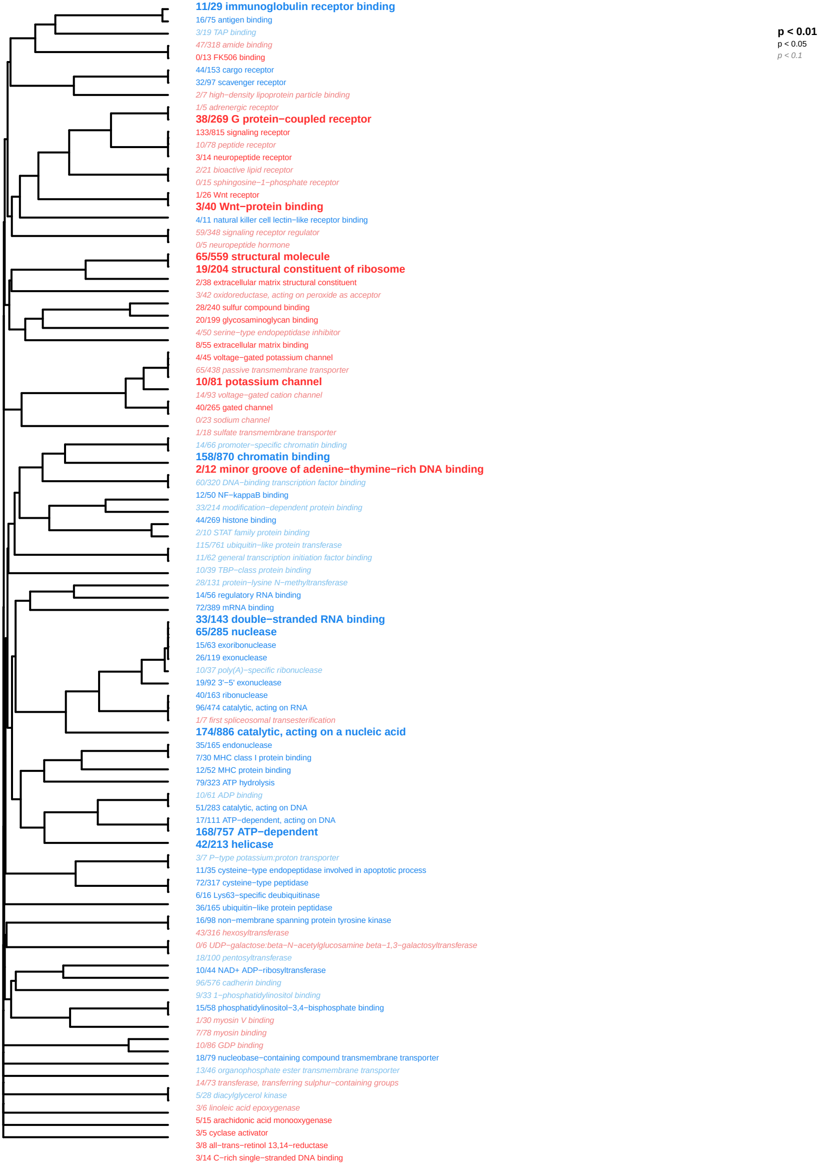
p < 0.01
p < 0.05
p < 0.1
11/29 immunoglobulin receptor binding
16/75 antigen binding
3/19 TAP binding
47/318 amide binding
0/13 FK506 binding
44/153 cargo receptor
32/97 scavenger receptor
2/7 high−density lipoprotein particle binding
1/5 adrenergic receptor
38/269 G protein−coupled receptor
133/815 signaling receptor
10/78 peptide receptor
3/14 neuropeptide receptor
2/21 bioactive lipid receptor
0/15 sphingosine−1−phosphate receptor
1/26 Wnt receptor
3/40 Wnt−protein binding
4/11 natural killer cell lectin−like receptor binding
59/348 signaling receptor regulator
0/5 neuropeptide hormone
65/559 structural molecule
19/204 structural constituent of ribosome
2/38 extracellular matrix structural constituent
3/42 oxidoreductase, acting on peroxide as acceptor
28/240 sulfur compound binding
20/199 glycosaminoglycan binding
4/50 serine−type endopeptidase inhibitor
8/55 extracellular matrix binding
4/45 voltage−gated potassium channel
65/438 passive transmembrane transporter
10/81 potassium channel
14/93 voltage−gated cation channel
40/265 gated channel
0/23 sodium channel
1/18 sulfate transmembrane transporter
14/66 promoter−specific chromatin binding
158/870 chromatin binding
2/12 minor groove of adenine−thymine−rich DNA binding
60/320 DNA−binding transcription factor binding
12/50 NF−kappaB binding
33/214 modification−dependent protein binding
44/269 histone binding
2/10 STAT family protein binding
115/761 ubiquitin−like protein transferase
11/62 general transcription initiation factor binding
10/39 TBP−class protein binding
28/131 protein−lysine N−methyltransferase
14/56 regulatory RNA binding
72/389 mRNA binding
33/143 double−stranded RNA binding
65/285 nuclease
15/63 exoribonuclease
26/119 exonuclease
10/37 poly(A)−specific ribonuclease
19/92 3'−5' exonuclease
40/163 ribonuclease
96/474 catalytic, acting on RNA
1/7 first spliceosomal transesterification
174/886 catalytic, acting on a nucleic acid
35/165 endonuclease
7/30 MHC class I protein binding
12/52 MHC protein binding
79/323 ATP hydrolysis
10/61 ADP binding
51/283 catalytic, acting on DNA
17/111 ATP−dependent, acting on DNA
168/757 ATP−dependent
42/213 helicase
3/7 P−type potassium:proton transporter
11/35 cysteine−type endopeptidase involved in apoptotic process
72/317 cysteine−type peptidase
6/16 Lys63−specific deubiquitinase
36/165 ubiquitin−like protein peptidase
16/98 non−membrane spanning protein tyrosine kinase
43/316 hexosyltransferase
0/6 UDP−galactose:beta−N−acetylglucosamine beta−1,3−galactosyltransferase
18/100 pentosyltransferase
10/44 NAD+ ADP−ribosyltransferase
96/576 cadherin binding
9/33 1−phosphatidylinositol binding
15/58 phosphatidylinositol−3,4−bisphosphate binding
1/30 myosin V binding
7/78 myosin binding
10/86 GDP binding
18/79 nucleobase−containing compound transmembrane transporter
13/46 organophosphate ester transmembrane transporter
14/73 transferase, transferring sulphur−containing groups
5/28 diacylglycerol kinase
3/6 linoleic acid epoxygenase
5/15 arachidonic acid monooxygenase
3/5 cyclase activator
3/8 all−trans−retinol 13,14−reductase
3/14 C−rich single−stranded DNA binding

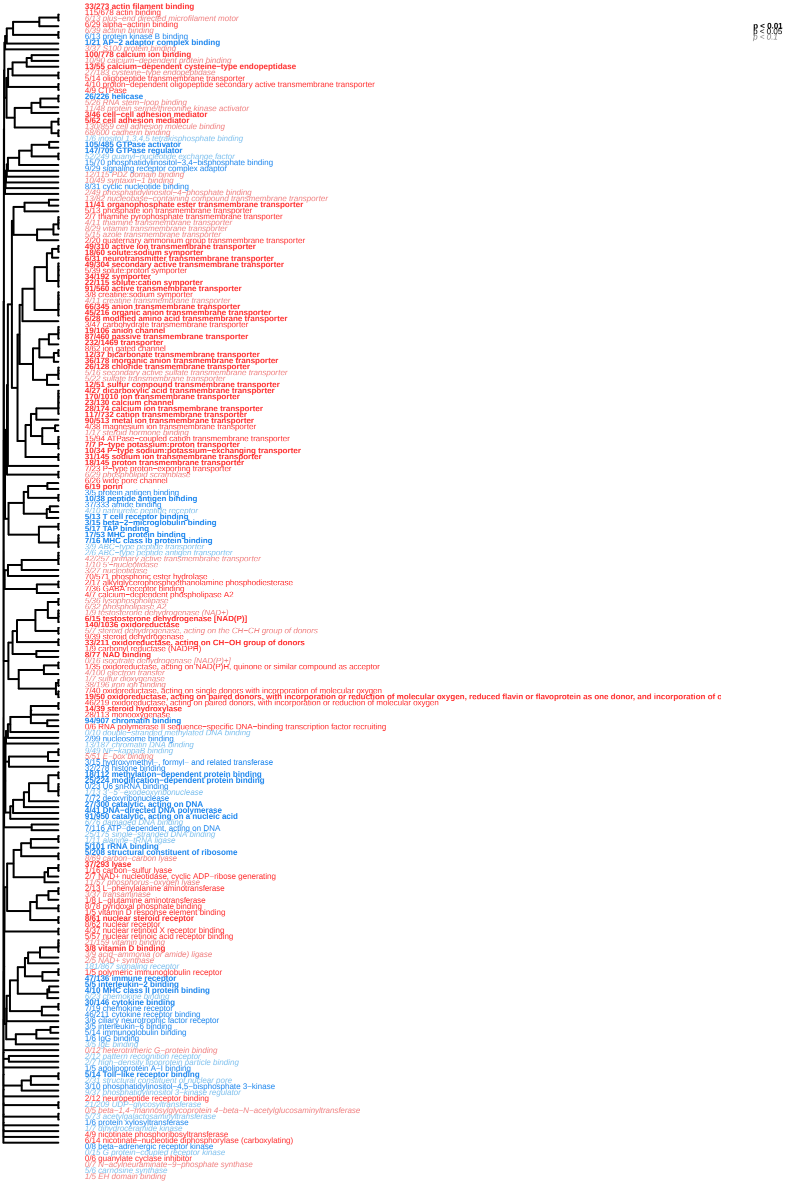
p < 0.01
p < 0.05
p < 0.1
33/273 actin filament binding
115/678 actin binding
6/13 plus−end directed microfilament motor
6/29 alpha−actinin binding
6/39 actinin binding
6/13 protein kinase B binding
1/21 AP−2 adaptor complex binding
3/37 S100 protein binding
100/778 calcium ion binding
10/90 calcium−dependent protein binding
13/55 calcium−dependent cysteine−type endopeptidase
27/183 cysteine−type endopeptidase
5/14 oligopeptide transmembrane transporter
4/10 proton−dependent oligopeptide secondary active transmembrane transporter
4/9 CTPase
26/226 helicase
5/26 RNA stem−loop binding
11/48 protein serine/threonine kinase activator
3/46 cell−cell adhesion mediator
5/62 cell adhesion mediator
130/859 cell adhesion molecule binding
68/600 cadherin binding
1/6 inositol 1,3,4,5 tetrakisphosphate binding
105/485 GTPase activator
147/709 GTPase regulator
52/249 guanyl−nucleotide exchange factor
15/70 phosphatidylinositol−3,4−bisphosphate binding
9/29 signaling receptor complex adaptor
12/115 PDZ domain binding
10/49 syntaxin−1 binding
8/31 cyclic nucleotide binding
2/49 phosphatidylinositol−4−phosphate binding
13/82 nucleobase−containing compound transmembrane transporter
11/41 organophosphate ester transmembrane transporter
5/13 phosphate ion transmembrane transporter
2/7 thiamine pyrophosphate transmembrane transporter
4/11 thiamine transmembrane transporter
8/29 vitamin transmembrane transporter
5/15 azole transmembrane transporter
2/20 quaternary ammonium group transmembrane transporter
49/310 active ion transmembrane transporter
18/60 solute:sodium symporter
6/31 neurotransmitter transmembrane transporter
49/304 secondary active transmembrane transporter
5/39 solute:proton symporter
34/192 symporter
22/115 solute:cation symporter
91/560 active transmembrane transporter
3/8 creatine:sodium symporter
4/11 creatine transmembrane transporter
66/345 anion transmembrane transporter
45/216 organic anion transmembrane transporter
6/28 modified amino acid transmembrane transporter
3/47 carbohydrate transmembrane transporter
19/106 anion channel
87/460 passive transmembrane transporter
232/1469 transporter
8/62 ion gated channel
12/37 bicarbonate transmembrane transporter
36/178 inorganic anion transmembrane transporter
26/128 chloride transmembrane transporter
5/16 secondary active sulfate transmembrane transporter
5/22 sulfate transmembrane transporter
12/51 sulfur compound transmembrane transporter
4/27 dicarboxylic acid transmembrane transporter
170/1010 ion transmembrane transporter
23/130 calcium channel
28/174 calcium ion transmembrane transporter
117/732 cation transmembrane transporter
90/513 metal ion transmembrane transporter
4/38 magnesium ion transmembrane transporter
1/17 steroid hormone binding
15/94 ATPase−coupled cation transmembrane transporter
7/7 P−type potassium:proton transporter
10/34 P−type sodium:potassium−exchanging transporter
31/145 sodium ion transmembrane transporter
18/145 proton transmembrane transporter
7/23 P−type proton−exporting transporter
6/29 phospholipid scramblase
6/26 wide pore channel
6/19 porin
3/5 protein antigen binding
10/38 peptide antigen binding
37/333 amide binding
4/10 natriuretic peptide receptor
5/13 T cell receptor binding
3/15 beta−2−microglobulin binding
5/17 TAP binding
17/53 MHC protein binding
7/16 MHC class Ib protein binding
3/9 ABC−type peptide transporter
2/6 ABC−type peptide antigen transporter
42/257 primary active transmembrane transporter
1/10 5'−nucleotidase
3/27 nucleotidase
70/571 phosphoric ester hydrolase
2/17 alkylglycerophosphoethanolamine phosphodiesterase
7/36 GABA receptor binding
4/7 calcium−dependent phospholipase A2
5/36 lysophospholipase
6/32 phospholipase A2
1/9 testosterone dehydrogenase (NAD+)
6/15 testosterone dehydrogenase [NAD(P)]
140/1036 oxidoreductase
5/7 steroid dehydrogenase, acting on the CH−CH group of donors
9/39 steroid dehydrogenase
33/211 oxidoreductase, acting on CH−OH group of donors
1/9 carbonyl reductase (NADPH)
8/77 NAD binding
0/16 isocitrate dehydrogenase [NAD(P)+]
1/35 oxidoreductase, acting on NAD(P)H, quinone or similar compound as acceptor
4/100 electron transfer
1/7 sulfur dioxygenase
38/196 iron ion binding
7/40 oxidoreductase, acting on single donors with incorporation of molecular oxygen
19/50 oxidoreductase, acting on paired donors, with incorporation or reduction of molecular oxygen, reduced flavin or flavoprotein as one donor, and incorporation of c
46/219 oxidoreductase, acting on paired donors, with incorporation or reduction of molecular oxygen
14/39 steroid hydroxylase
28/113 monooxygenase
94/907 chromatin binding
0/6 RNA polymerase II sequence−specific DNA−binding transcription factor recruiting
0/10 double−stranded methylated DNA binding
2/99 nucleosome binding
13/187 chromatin DNA binding
9/49 NF−kappaB binding
5/51 E−box binding
3/15 hydroxymethyl−, formyl− and related transferase
32/278 histone binding
18/112 methylation−dependent protein binding
25/224 modification−dependent protein binding
0/23 U6 snRNA binding
1/13 3'−5'−exodeoxyribonuclease
7/72 deoxyribonuclease
27/300 catalytic, acting on DNA
4/41 DNA−directed DNA polymerase
91/950 catalytic, acting on a nucleic acid
6/76 damaged DNA binding
7/116 ATP−dependent, acting on DNA
25/175 single−stranded DNA binding
1/11 alanine−tRNA ligase
5/101 rRNA binding
5/208 structural constituent of ribosome
8/69 carbon−carbon lyase
37/293 lyase
1/16 carbon−sulfur lyase
2/7 NAD+ nucleotidase, cyclic ADP−ribose generating
11/57 phosphorus−oxygen lyase
2/13 L−phenylalanine aminotransferase
3/37 transaminase
1/8 L−glutamine aminotransferase
8/78 pyridoxal phosphate binding
1/5 vitamin D response element binding
8/61 nuclear steroid receptor
8/62 nuclear receptor
4/37 nuclear retinoid X receptor binding
5/57 nuclear retinoic acid receptor binding
21/159 vitamin binding
3/8 vitamin D binding
3/9 acid−ammonia (or amide) ligase
2/5 NAD+ synthase
181/867 signaling receptor
1/5 polymeric immunoglobulin receptor
47/136 immune receptor
5/5 interleukin−2 binding
4/10 MHC class II protein binding
6/23 chemokine binding
30/146 cytokine binding
7/19 chemokine receptor
46/211 cytokine receptor binding
3/6 ciliary neurotrophic factor receptor
3/5 interleukin−6 binding
5/14 immunoglobulin binding
1/6 IgG binding
3/5 IgE binding
0/12 heterotrimeric G−protein binding
2/12 pattern recognition receptor
2/7 high−density lipoprotein particle binding
1/5 apolipoprotein A−I binding
5/14 Toll−like receptor binding
2/31 structural constituent of nuclear pore
3/10 phosphatidylinositol−4,5−bisphosphate 3−kinase
9/37 phosphatidylinositol 3−kinase regulator
2/12 neuropeptide receptor binding
21/209 UDP−glycosyltransferase
0/5 beta−1,4−mannosylglycoprotein 4−beta−N−acetylglucosaminyltransferase
5/73 acetylgalactosaminyltransferase
1/6 protein xylosyltransferase
1/7 dihydroceramide kinase
4/9 nicotinate phosphoribosyltransferase
6/14 nicotinate−nucleotide diphosphorylase (carboxylating)
0/8 beta−adrenergic receptor kinase
0/15 G protein−coupled receptor kinase
0/6 guanylate cyclase inhibitor
0/7 N−acylneuraminate−9−phosphate synthase
5/6 carnosine synthase
1/5 EH domain binding

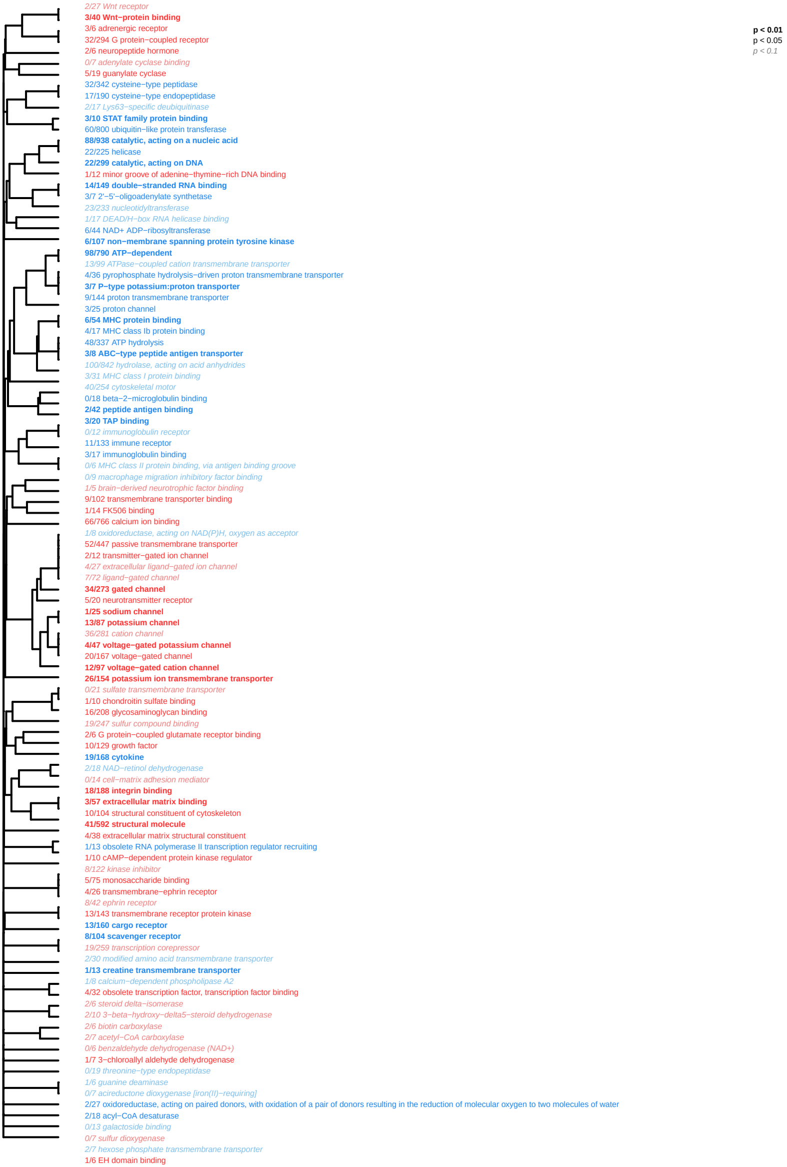
p < 0.01
p < 0.05
p < 0.1
2/27 Wnt receptor
3/40 Wnt−protein binding
3/6 adrenergic receptor
32/294 G protein−coupled receptor
2/6 neuropeptide hormone
0/7 adenylate cyclase binding
5/19 guanylate cyclase
32/342 cysteine−type peptidase
17/190 cysteine−type endopeptidase
2/17 Lys63−specific deubiquitinase
3/10 STAT family protein binding
60/800 ubiquitin−like protein transferase
88/938 catalytic, acting on a nucleic acid
22/225 helicase
22/299 catalytic, acting on DNA
1/12 minor groove of adenine−thymine−rich DNA binding
14/149 double−stranded RNA binding
3/7 2'−5'−oligoadenylate synthetase
23/233 nucleotidyltransferase
1/17 DEAD/H−box RNA helicase binding
6/44 NAD+ ADP−ribosyltransferase
6/107 non−membrane spanning protein tyrosine kinase
98/790 ATP−dependent
13/99 ATPase−coupled cation transmembrane transporter
4/36 pyrophosphate hydrolysis−driven proton transmembrane transporter
3/7 P−type potassium:proton transporter
9/144 proton transmembrane transporter
3/25 proton channel
6/54 MHC protein binding
4/17 MHC class Ib protein binding
48/337 ATP hydrolysis
3/8 ABC−type peptide antigen transporter
100/842 hydrolase, acting on acid anhydrides
3/31 MHC class I protein binding
40/254 cytoskeletal motor
0/18 beta−2−microglobulin binding
2/42 peptide antigen binding
3/20 TAP binding
0/12 immunoglobulin receptor
11/133 immune receptor
3/17 immunoglobulin binding
0/6 MHC class II protein binding, via antigen binding groove
0/9 macrophage migration inhibitory factor binding
1/5 brain−derived neurotrophic factor binding
9/102 transmembrane transporter binding
1/14 FK506 binding
66/766 calcium ion binding
1/8 oxidoreductase, acting on NAD(P)H, oxygen as acceptor
52/447 passive transmembrane transporter
2/12 transmitter−gated ion channel
4/27 extracellular ligand−gated ion channel
7/72 ligand−gated channel
34/273 gated channel
5/20 neurotransmitter receptor
1/25 sodium channel
13/87 potassium channel
36/281 cation channel
4/47 voltage−gated potassium channel
20/167 voltage−gated channel
12/97 voltage−gated cation channel
26/154 potassium ion transmembrane transporter
0/21 sulfate transmembrane transporter
1/10 chondroitin sulfate binding
16/208 glycosaminoglycan binding
19/247 sulfur compound binding
2/6 G protein−coupled glutamate receptor binding
10/129 growth factor
19/168 cytokine
2/18 NAD−retinol dehydrogenase
0/14 cell−matrix adhesion mediator
18/188 integrin binding
3/57 extracellular matrix binding
10/104 structural constituent of cytoskeleton
41/592 structural molecule
4/38 extracellular matrix structural constituent
1/13 obsolete RNA polymerase II transcription regulator recruiting
1/10 cAMP−dependent protein kinase regulator
8/122 kinase inhibitor
5/75 monosaccharide binding
4/26 transmembrane−ephrin receptor
8/42 ephrin receptor
13/143 transmembrane receptor protein kinase
13/160 cargo receptor
8/104 scavenger receptor
19/259 transcription corepressor
2/30 modified amino acid transmembrane transporter
1/13 creatine transmembrane transporter
1/8 calcium−dependent phospholipase A2
4/32 obsolete transcription factor, transcription factor binding
2/6 steroid delta−isomerase
2/10 3−beta−hydroxy−delta5−steroid dehydrogenase
2/6 biotin carboxylase
2/7 acetyl−CoA carboxylase
0/6 benzaldehyde dehydrogenase (NAD+)
1/7 3−chloroallyl aldehyde dehydrogenase
0/19 threonine−type endopeptidase
1/6 guanine deaminase
0/7 acireductone dioxygenase [iron(II)−requiring]
2/27 oxidoreductase, acting on paired donors, with oxidation of a pair of donors resulting in the reduction of molecular oxygen to two molecules of water
2/18 acyl−CoA desaturase
0/13 galactoside binding
0/7 sulfur dioxygenase
2/7 hexose phosphate transmembrane transporter
1/6 EH domain binding

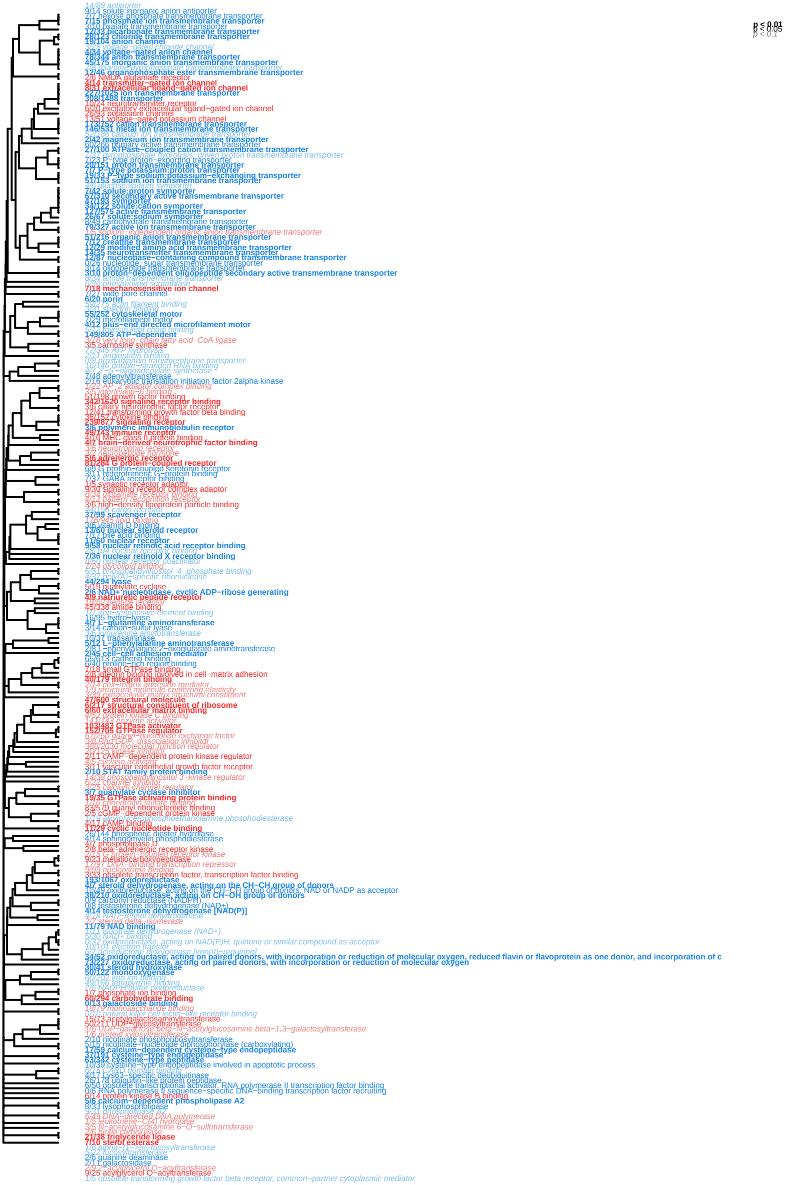
p < 0.01
p < 0.05
p < 0.1
14/89 antiporter
9/14 solute:inorganic anion antiporter
7/7 hexose phosphate transmembrane transporter
7/15 phosphate ion transmembrane transporter
3/10 oxalate transmembrane transporter
12/33 bicarbonate transmembrane transporter
28/123 chloride transmembrane transporter
19/104 anion channel
5/25 voltage−gated chloride channel
4/34 voltage−gated anion channel
78/344 anion transmembrane transporter
45/175 inorganic anion transmembrane transporter
2/5 glycerol−3−phosphate transmembrane transporter
12/46 organophosphate ester transmembrane transporter
2/6 NMDA glutamate receptor
4/14 transmitter−gated ion channel
8/31 extracellular ligand−gated ion channel
227/1025 ion transmembrane transporter
308/1488 transporter
10/24 neurotransmitter receptor
6/20 excitatory extracellular ligand−gated ion channel
26/93 potassium channel
13/51 voltage−gated potassium channel
173/752 cation transmembrane transporter
146/531 metal ion transmembrane transporter
52/186 calcium ion transmembrane transporter
2/42 magnesium ion transmembrane transporter
60/285 primary active transmembrane transporter
27/100 ATPase−coupled cation transmembrane transporter
2/37 pyrophosphate hydrolysis−driven proton transmembrane transporter
7/23 P−type proton−exporting transporter
20/151 proton transmembrane transporter
7/7 P−type potassium:proton transporter
19/33 P−type sodium:potassium−exchanging transporter
51/153 sodium ion transmembrane transporter
4/6 glucose:sodium symporter
7/42 solute:proton symporter
67/310 secondary active transmembrane transporter
47/193 symporter
33/122 solute:cation symporter
127/575 active transmembrane transporter
26/67 solute:sodium symporter
8/49 carbohydrate transmembrane transporter
79/327 active ion transmembrane transporter
1/5 sodium−independent organic anion transmembrane transporter
51/216 organic anion transmembrane transporter
7/12 creatine transmembrane transporter
12/29 modified amino acid transmembrane transporter
13/35 neurotransmitter transmembrane transporter
12/87 nucleobase−containing compound transmembrane transporter
0/26 nucleotide−sugar transmembrane transporter
3/14 oligopeptide transmembrane transporter
3/10 proton−dependent oligopeptide secondary active transmembrane transporter
8/34 amide transmembrane transporter
6/30 phospholipid scramblase
7/18 mechanosensitive ion channel
7/27 wide pore channel
6/20 porin
59/275 actin filament binding
3/21 spectrin binding
55/252 cytoskeletal motor
7/29 microfilament motor
4/12 plus−end directed microfilament motor
5/11 myosin light chain binding
149/805 ATP−dependent
3/18 very long−chain fatty acid−CoA ligase
3/5 carnosine synthase
77/345 ATP hydrolysis
2/11 angiostatin binding
0/8 prostaglandin transmembrane transporter
16/146 double−stranded RNA binding
4/7 2'−5'−oligoadenylate synthetase
7/48 adenylyltransferase
2/16 eukaryotic translation initiation factor 2alpha kinase
1/22 AP−2 adaptor complex binding
3/5 interleukin−6 binding
51/198 growth factor binding
342/1620 signaling receptor binding
3/6 ciliary neurotrophic factor receptor
12/41 transforming growth factor beta binding
36/152 cytokine binding
239/877 signaling receptor
3/6 polymeric immunoglobulin receptor
49/143 immune receptor
4/10 MHC class II protein binding
4/7 brain−derived neurotrophic factor binding
3/6 neurotrophin receptor
3/7 neuropeptide hormone
5/6 adrenergic receptor
81/284 G protein−coupled receptor
6/9 G protein−coupled serotonin receptor
3/11 heterotrimeric G−protein binding
7/37 GABA receptor binding
1/5 synaptic receptor adaptor
9/30 signaling receptor complex adaptor
9/34 glutamate receptor binding
4/12 pattern recognition receptor
3/6 high−density lipoprotein particle binding
44/158 cargo receptor
37/99 scavenger receptor
176/945 lipid binding
3/6 vitamin D binding
13/60 nuclear steroid receptor
7/17 bile acid binding
11/60 nuclear receptor
9/58 nuclear retinoic acid receptor binding
28/194 nuclear receptor binding
7/36 nuclear retinoid X receptor binding
8/60 nuclear receptor coactivator
7/24 glycolipid binding
6/51 phosphatidylinositol−4−phosphate binding
4/32 poly(A)−specific ribonuclease
44/294 lyase
5/19 guanylate cyclase
2/6 NAD+ nucleotidase, cyclic ADP−ribose generating
4/9 natriuretic peptide receptor
10/62 peptide receptor
45/338 amide binding
1/7 iron−responsive element binding
16/95 hydro−lyase
4/7 L−glutamine aminotransferase
3/14 carbon−sulfur lyase
3/6 kynurenine aminotransferase
10/37 transaminase
5/12 L−phenylalanine aminotransferase
2/8 L−phenylalanine:2−oxoglutarate aminotransferase
2/45 cell−cell adhesion mediator
65/313 cadherin binding
6/40 proline−rich region binding
7/18 small GTPase binding
2/8 integrin binding involved in cell−matrix adhesion
40/179 integrin binding
2/14 cell−matrix adhesion mediator
1/9 structural molecule conferring elasticity
3/39 extracellular matrix structural constituent
47/600 structural molecule
6/217 structural constituent of ribosome
6/60 extracellular matrix binding
4/52 protein kinase C binding
141/743 enzyme activator
103/483 GTPase activator
152/705 GTPase regulator
67/259 guanyl−nucleotide exchange factor
3/8 Rho GDP−dissociation inhibitor
388/2030 molecular function regulator
38/125 kinase inhibitor
2/11 cAMP−dependent protein kinase regulator
4/5 cyclase activator
3/11 vascular endothelial growth factor receptor
2/10 STAT family protein binding
14/38 phosphatidylinositol 3−kinase regulator
6/22 channel inhibitor
3/25 calcium channel regulator
3/7 guanylate cyclase inhibitor
19/35 GTPase activating protein binding
1/10 chondroitin sulfate binding
83/579 guanyl ribonucleotide binding
2/5 cGMP−dependent protein kinase
1/19 alkylglycerophosphoethanolamine phosphodiesterase
4/17 cAMP binding
11/29 cyclic nucleotide binding
26/144 phosphoric diester hydrolase
4/14 sphingomyelin phosphodiesterase
4/7 phospholipase D
2/8 beta−adrenergic receptor kinase
2/15 G protein−coupled receptor kinase
9/23 metallocarboxypeptidase
17/97 DNA−binding transcription repressor
9/99 nucleosome binding
3/33 obsolete transcription factor, transcription factor binding
193/1067 oxidoreductase
4/7 steroid dehydrogenase, acting on the CH−CH group of donors
10/49 oxidoreductase, acting on the CH−CH group of donors, NAD or NADP as acceptor
38/210 oxidoreductase, acting on CH−OH group of donors
0/9 carbonyl reductase (NADPH)
0/8 testosterone dehydrogenase (NAD+)
4/14 testosterone dehydrogenase [NAD(P)]
4/18 NAD−retinol dehydrogenase
2/7 steroid delta−isomerase
11/79 NAD binding
1/7 isocitrate dehydrogenase (NAD+)
5/30 NAD+ binding
0/37 oxidoreductase, acting on NAD(P)H, quinone or similar compound as acceptor
10/101 electron transfer
6/7 acireductone dioxygenase [iron(II)−requiring]
34/52 oxidoreductase, acting on paired donors, with incorporation or reduction of molecular oxygen, reduced flavin or flavoprotein as one donor, and incorporation of o
73/227 oxidoreductase, acting on paired donors, with incorporation or reduction of molecular oxygen
30/41 steroid hydroxylase
50/122 monooxygenase
60/202 iron ion binding
49/166 tetrapyrrole binding
2/6 NADPH−sulfur oxidoreductase
1/7 phosphate ion binding
60/294 carbohydrate binding
0/13 galactoside binding
18/79 monosaccharide binding
0/10 natural killer cell lectin−like receptor binding
15/73 acetylgalactosaminyltransferase
50/211 UDP−glycosyltransferase
1/6 UDP−galactose:beta−N−acetylglucosamine beta−1,3−galactosyltransferase
1/6 protein xylosyltransferase
2/10 nicotinate phosphoribosyltransferase
5/15 nicotinate−nucleotide diphosphorylase (carboxylating)
17/59 calcium−dependent cysteine−type endopeptidase
37/191 cysteine−type endopeptidase
63/342 cysteine−type peptidase
10/39 cysteine−type endopeptidase involved in apoptotic process
6/19 CARD domain binding
4/17 Lys63−specific deubiquitinase
26/178 ubiquitin−like protein peptidase
6/50 obsolete transcriptional activator, RNA polymerase II transcription factor binding
0/6 RNA polymerase II sequence−specific DNA−binding transcription factor recruiting
6/14 protein kinase B binding
5/6 calcium−dependent phospholipase A2
8/33 lysophospholipase
9/31 phospholipase A2
6/49 DNA−directed DNA polymerase
1/5 leukotriene−C(4) hydrolase
3/5 N−acetylglucosamine 6−O−sulfotransferase
2/6 biotin carboxylase
21/38 triglyceride lipase
7/10 sterol esterase
1/6 alpha−(1−>6)−fucosyltransferase
5/22 fucosyltransferase
2/6 guanine deaminase
2/11 galactosidase
0/9 2−acylglycerol O−acyltransferase
9/25 acylglycerol O−acyltransferase
1/5 obsolete transforming growth factor beta receptor, common−partner cytoplasmic mediator
