# Supplementary material for: Limitation of amino acid availability by bacterial populations during enhanced colitis in IBD mouse model
Source: mSystems. 2023 Nov 1;8(6):e00703-23. doi: 10.1128/msystems.00703-23 (PMC10746178; doi:10.1128/msystems.00703-23)
Supplement: Figure S3 — Phylogenetic trees of the 7 MAGs of interest. [file msystems.00703-23-s0003.pdf]

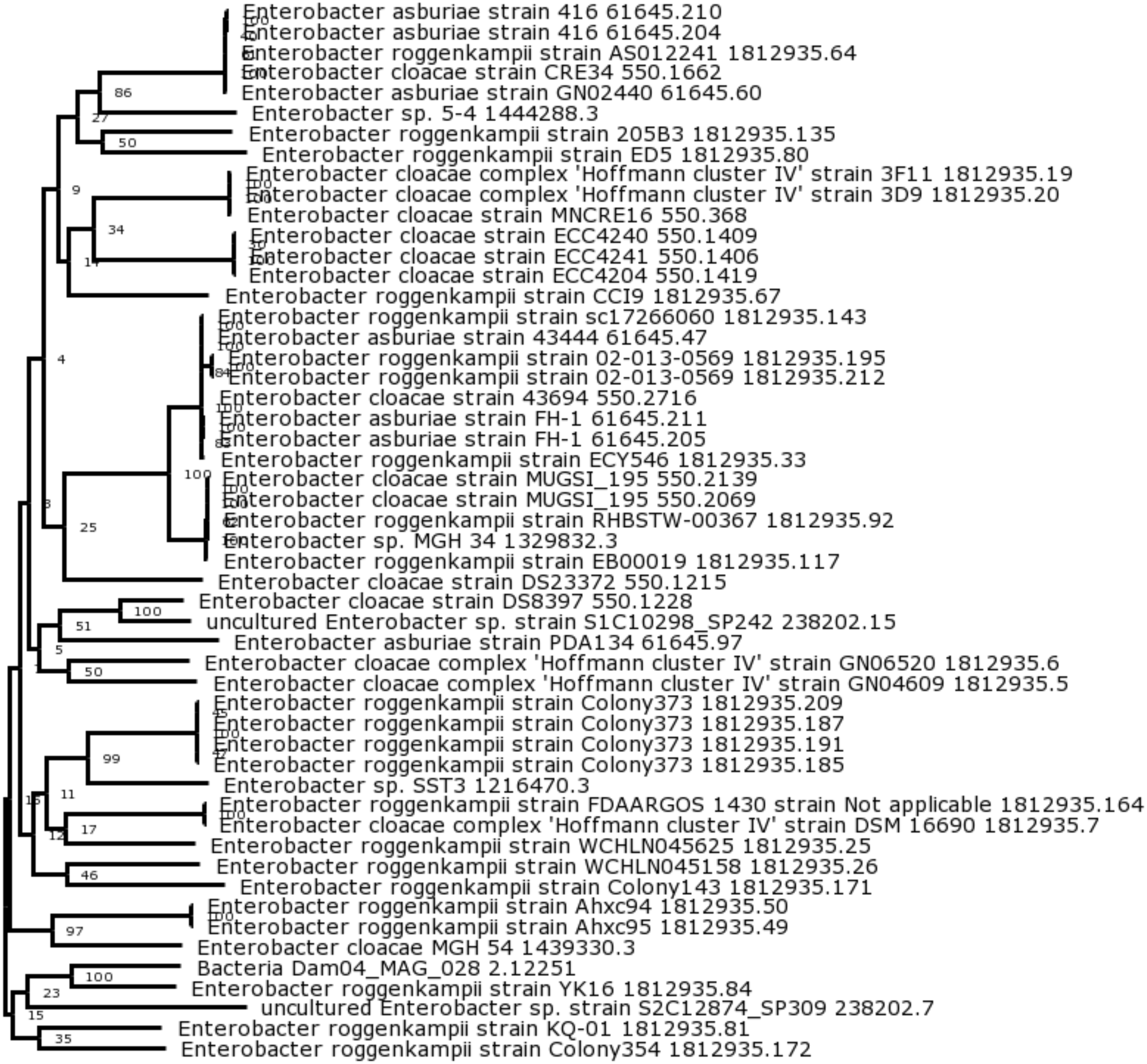

Supplementary Figure S3. Phylogenetic tree of the highest related strains of 7 MAGs of interest.

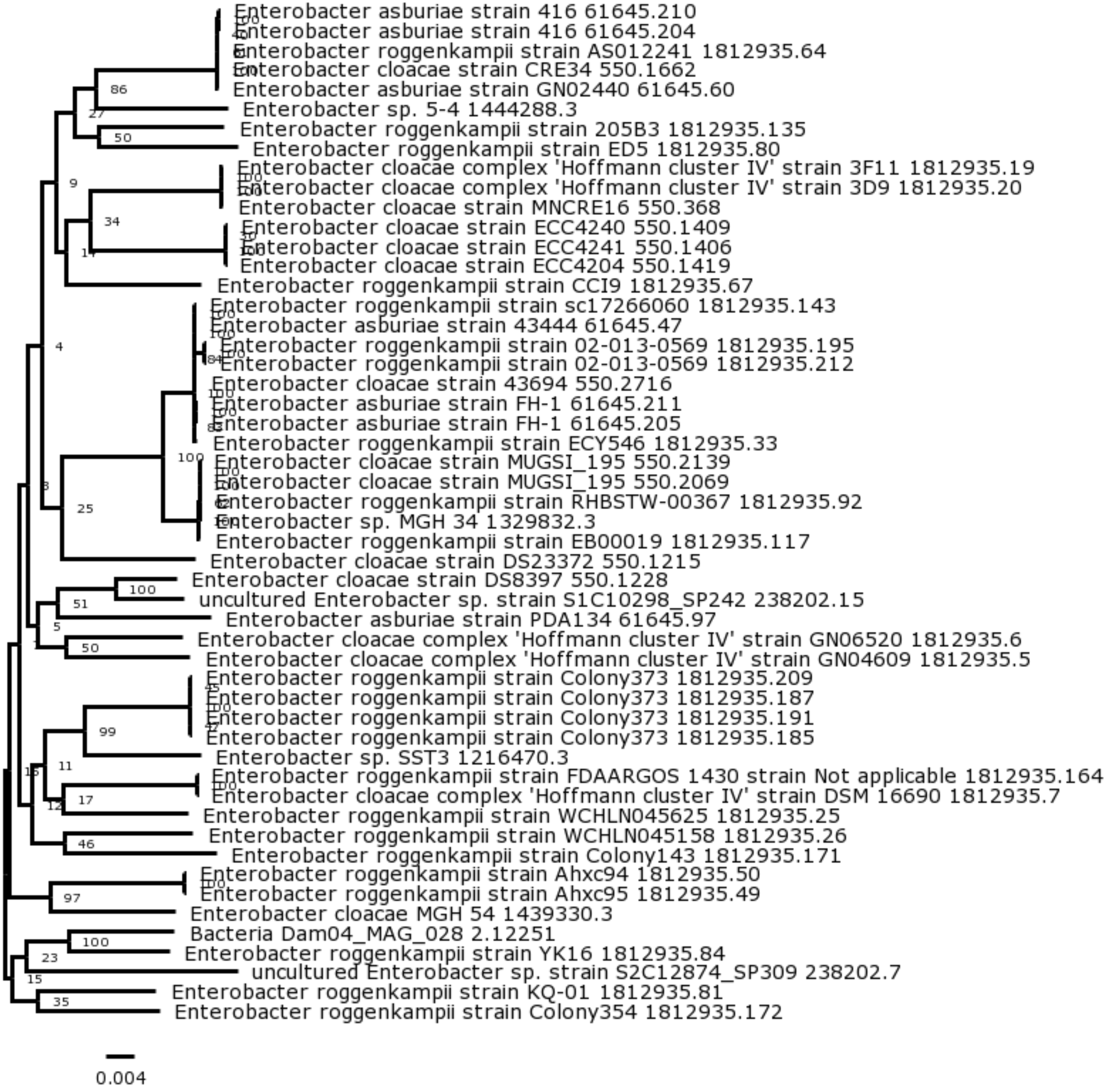
Enterobacter asburiae strain 416 61645.210
Enterobacter asburiae strain 416 61645.204
Enterobacter roggenkampii strain AS012241 1812935.64
Enterobacter cloacae strain CRE34 550.1662
Enterobacter asburiae strain GN02440 61645.60
Enterobacter sp. 5-4 1444288.3
Enterobacter roggenkampii strain 205B3 1812935.135
Enterobacter roggenkampii strain ED5 1812935.80
Enterobacter cloacae complex 'Hoffmann cluster IV' strain 3F11 1812935.19
Enterobacter cloacae complex 'Hoffmann cluster IV' strain 3D9 1812935.20
Enterobacter cloacae strain MNCRE16 550.368
Enterobacter cloacae strain ECC4240 550.1409
Enterobacter cloacae strain ECC4241 550.1406
Enterobacter cloacae strain ECC4204 550.1419
Enterobacter roggenkampii strain CCI9 1812935.67
Enterobacter roggenkampii strain sc17266060 1812935.143
Enterobacter asburiae strain 43444 61645.47
Enterobacter roggenkampii strain 02-013-0569 1812935.195
Enterobacter roggenkampii strain 02-013-0569 1812935.212
Enterobacter cloacae strain 43694 550.2716
Enterobacter asburiae strain FH-1 61645.211
Enterobacter asburiae strain FH-1 61645.205
Enterobacter roggenkampii strain ECY546 1812935.33
Enterobacter cloacae strain MUGSI_195 550.2139
Enterobacter cloacae strain MUGSI_195 550.2069
Enterobacter roggenkampii strain RHBSTW-00367 1812935.92
Enterobacter sp. MGH 34 1329832.3
Enterobacter roggenkampii strain EB00019 1812935.117
Enterobacter cloacae strain DS23372 550.1215
Enterobacter cloacae strain DS8397 550.1228
uncultured Enterobacter sp. strain S1C10298_SP242 238202.15
Enterobacter asburiae strain PDA134 61645.97
Enterobacter cloacae complex 'Hoffmann cluster IV' strain GN06520 1812935.6
Enterobacter cloacae complex 'Hoffmann cluster IV' strain GN04609 1812935.5
Enterobacter roggenkampii strain Colony373 1812935.209
Enterobacter roggenkampii strain Colony373 1812935.187
Enterobacter roggenkampii strain Colony373 1812935.191
Enterobacter roggenkampii strain Colony373 1812935.185
Enterobacter sp. SST3 1216470.3
Enterobacter roggenkampii strain FDAARGOS 1430 strain Not applicable 1812935.164
Enterobacter cloacae complex 'Hoffmann cluster IV' strain DSM 16690 1812935.7
Enterobacter roggenkampii strain WCHLN045625 1812935.25
Enterobacter roggenkampii strain WCHLN045158 1812935.26
Enterobacter roggenkampii strain Colony143 1812935.171
Enterobacter roggenkampii strain Ahxc94 1812935.50
Enterobacter roggenkampii strain Ahxc95 1812935.49
Enterobacter cloacae MGH 54 1439330.3
Bacteria Dam04_MAG_028 2.12251
Enterobacter roggenkampii strain YK16 1812935.84
uncultured Enterobacter sp. strain S2C12874_SP309 238202.7
Enterobacter roggenkampii strain KQ-01 1812935.81
Enterobacter roggenkampii strain Colony354 1812935.172
0.004

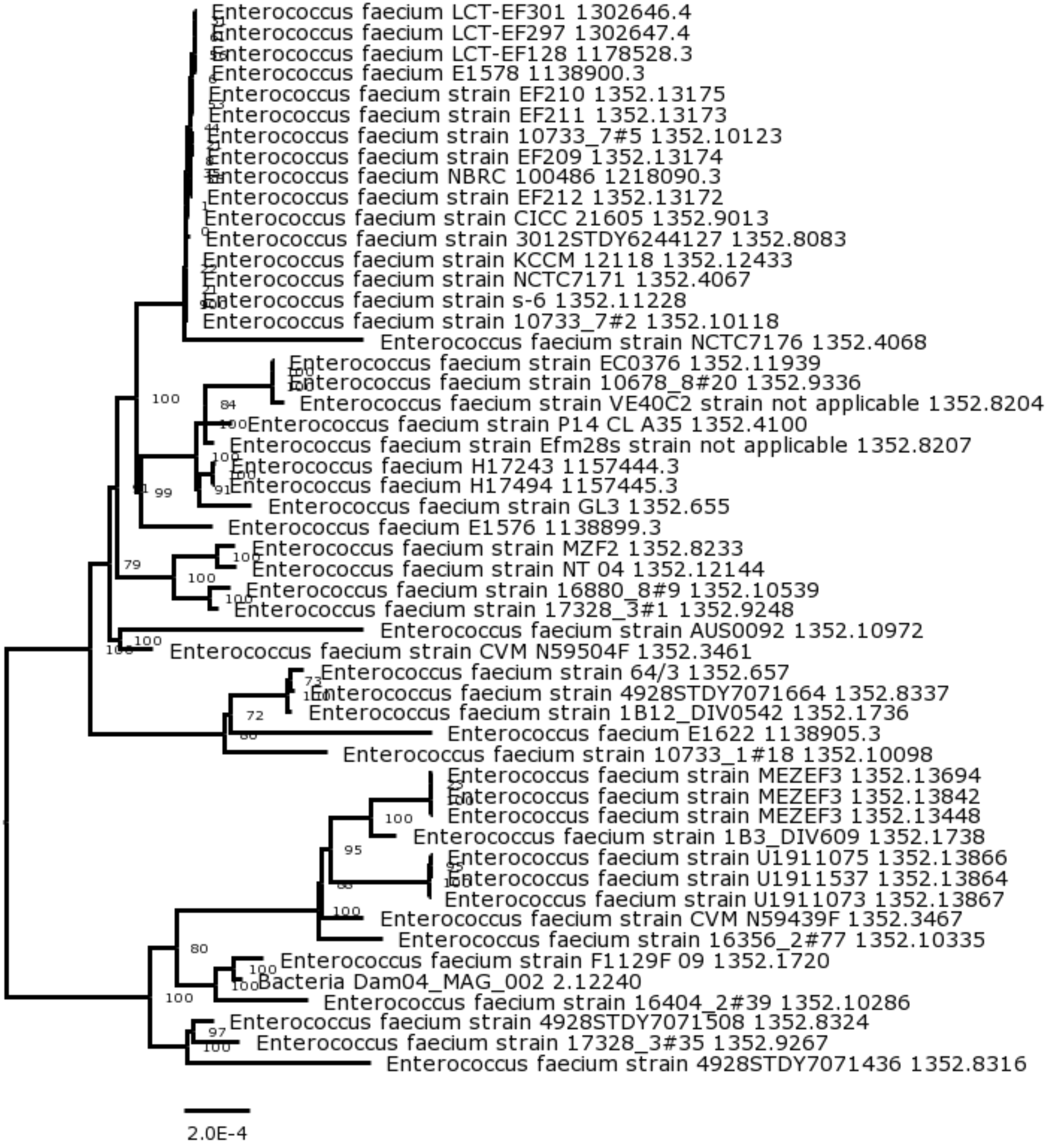
Enterococcus faecium LCT-EF301 1302646.4
Enterococcus faecium LCT-EF297 1302647.4
Enterococcus faecium LCT-EF128 1178528.3
Enterococcus faecium E1578 1138900.3
Enterococcus faecium strain EF210 1352.13175
Enterococcus faecium strain EF211 1352.13173
Enterococcus faecium strain 10733_7#5 1352.10123
Enterococcus faecium strain EF209 1352.13174
Enterococcus faecium NBRC 100486 1218090.3
Enterococcus faecium strain EF212 1352.13172
Enterococcus faecium strain CICC 21605 1352.9013
Enterococcus faecium strain 3012STDY6244127 1352.8083
Enterococcus faecium strain KCCM 12118 1352.12433
Enterococcus faecium strain NCTC7171 1352.4067
Enterococcus faecium strain s-6 1352.11228
Enterococcus faecium strain 10733_7#2 1352.10118
Enterococcus faecium strain NCTC7176 1352.4068
Enterococcus faecium strain EC0376 1352.11939
Enterococcus faecium strain 10678_8#20 1352.9336
Enterococcus faecium strain VE40C2 strain not applicable 1352.8204
Enterococcus faecium strain P14 CL A35 1352.4100
Enterococcus faecium strain Efm28s strain not applicable 1352.8207
Enterococcus faecium H17243 1157444.3
Enterococcus faecium H17494 1157445.3
Enterococcus faecium strain GL3 1352.655
Enterococcus faecium E1576 1138899.3
Enterococcus faecium strain MZF2 1352.8233
Enterococcus faecium strain NT 04 1352.12144
Enterococcus faecium strain 16880_8#9 1352.10539
Enterococcus faecium strain 17328_3#1 1352.9248
Enterococcus faecium strain AUS0092 1352.10972
Enterococcus faecium strain CVM N59504F 1352.3461
Enterococcus faecium strain 64/3 1352.657
Enterococcus faecium strain 4928STDY7071664 1352.8337
Enterococcus faecium strain 1B12_DIV0542 1352.1736
Enterococcus faecium E1622 1138905.3
Enterococcus faecium strain 10733_1#18 1352.10098
Enterococcus faecium strain MEZEF3 1352.13694
Enterococcus faecium strain MEZEF3 1352.13842
Enterococcus faecium strain MEZEF3 1352.13448
Enterococcus faecium strain 1B3_DIV609 1352.1738
Enterococcus faecium strain U1911075 1352.13866
Enterococcus faecium strain U1911537 1352.13864
Enterococcus faecium strain U1911073 1352.13867
Enterococcus faecium strain CVM N59439F 1352.3467
Enterococcus faecium strain 16356_2#77 1352.10335
Enterococcus faecium strain F1129F 09 1352.1720
Bacteria Dam04_MAG_002 2.12240
Enterococcus faecium strain 16404_2#39 1352.10286
Enterococcus faecium strain 4928STDY7071508 1352.8324
Enterococcus faecium strain 17328_3#35 1352.9267
Enterococcus faecium strain 4928STDY7071436 1352.8316
2.0E-4

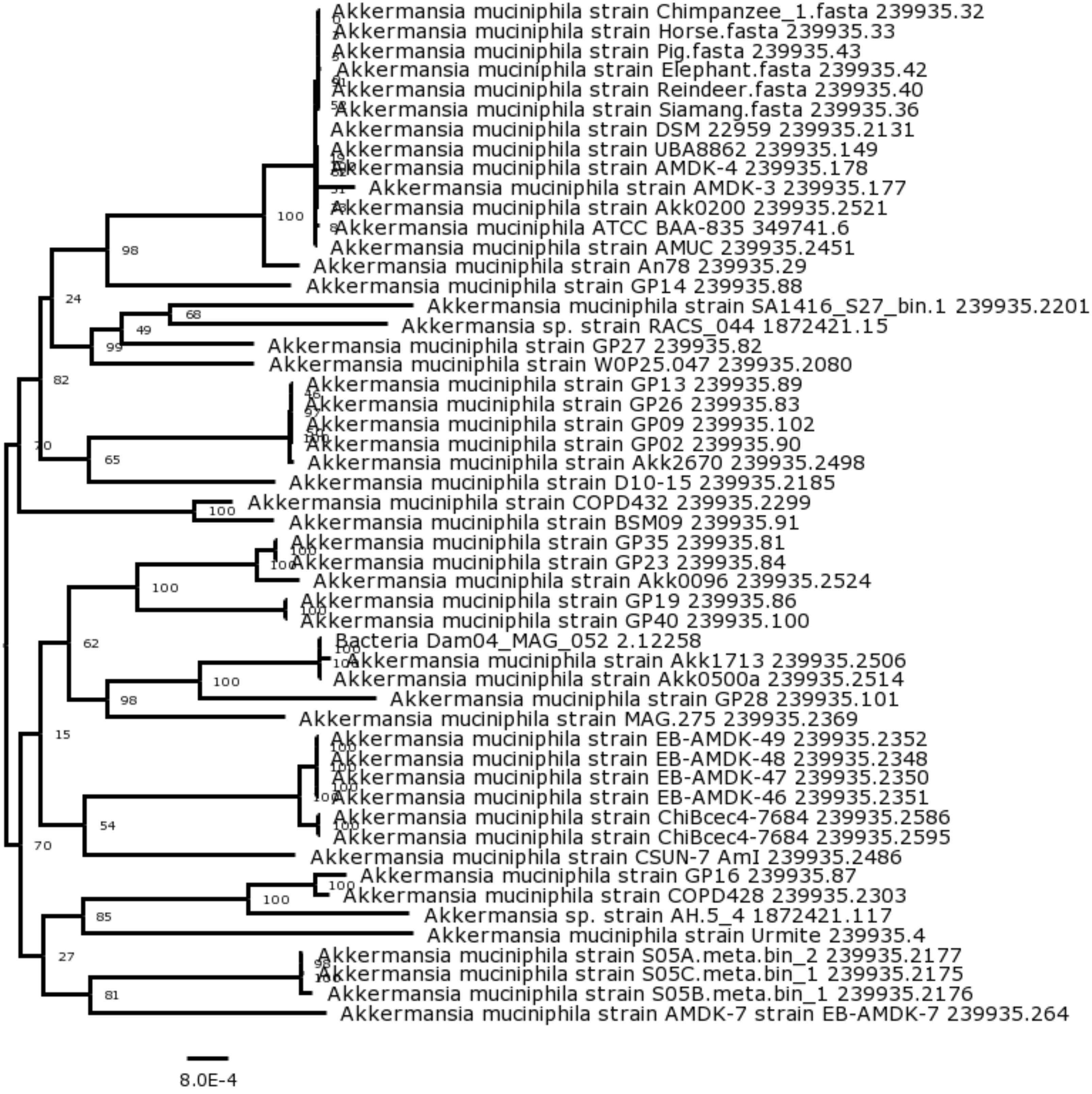
Akkermansia muciniphila strain Chimpanzee_1.fasta 239935.32
Akkermansia muciniphila strain Horse.fasta 239935.33
Akkermansia muciniphila strain Pig.fasta 239935.43
Akkermansia muciniphila strain Elephant.fasta 239935.42
Akkermansia muciniphila strain Reindeer.fasta 239935.40
Akkermansia muciniphila strain Siamang.fasta 239935.36
Akkermansia muciniphila strain DSM 22959 239935.2131
Akkermansia muciniphila strain UBA8862 239935.149
Akkermansia muciniphila strain AMDK-4 239935.178
Akkermansia muciniphila strain AMDK-3 239935.177
Akkermansia muciniphila strain Akk0200 239935.2521
Akkermansia muciniphila ATCC BAA-835 349741.6
Akkermansia muciniphila strain AMUC 239935.2451
Akkermansia muciniphila strain An78 239935.29
Akkermansia muciniphila strain GP14 239935.88
Akkermansia muciniphila strain SA1416_S27_bin.1 239935.2201
Akkermansia sp. strain RACS_044 1872421.15
Akkermansia muciniphila strain GP27 239935.82
Akkermansia muciniphila strain W0P25.047 239935.2080
Akkermansia muciniphila strain GP13 239935.89
Akkermansia muciniphila strain GP26 239935.83
Akkermansia muciniphila strain GP09 239935.102
Akkermansia muciniphila strain GP02 239935.90
Akkermansia muciniphila strain Akk2670 239935.2498
Akkermansia muciniphila strain D10-15 239935.2185
Akkermansia muciniphila strain COPD432 239935.2299
Akkermansia muciniphila strain BSM09 239935.91
Akkermansia muciniphila strain GP35 239935.81
Akkermansia muciniphila strain GP23 239935.84
Akkermansia muciniphila strain Akk0096 239935.2524
Akkermansia muciniphila strain GP19 239935.86
Akkermansia muciniphila strain GP40 239935.100
Bacteria Dam04_MAG_052 2.12258
Akkermansia muciniphila strain Akk1713 239935.2506
Akkermansia muciniphila strain Akk0500a 239935.2514
Akkermansia muciniphila strain GP28 239935.101
Akkermansia muciniphila strain MAG.275 239935.2369
Akkermansia muciniphila strain EB-AMDK-49 239935.2352
Akkermansia muciniphila strain EB-AMDK-48 239935.2348
Akkermansia muciniphila strain EB-AMDK-47 239935.2350
Akkermansia muciniphila strain EB-AMDK-46 239935.2351
Akkermansia muciniphila strain ChiBcec4-7684 239935.2586
Akkermansia muciniphila strain ChiBcec4-7684 239935.2595
Akkermansia muciniphila strain CSUN-7 AmI 239935.2486
Akkermansia muciniphila strain GP16 239935.87
Akkermansia muciniphila strain COPD428 239935.2303
Akkermansia sp. strain AH.5_4 1872421.117
Akkermansia muciniphila strain Urmite 239935.4
Akkermansia muciniphila strain S05A.meta.bin_2 239935.2177
Akkermansia muciniphila strain S05C.meta.bin_1 239935.2175
Akkermansia muciniphila strain S05B.meta.bin_1 239935.2176
Akkermansia muciniphila strain AMDK-7 strain EB-AMDK-7 239935.264
8.0E-4

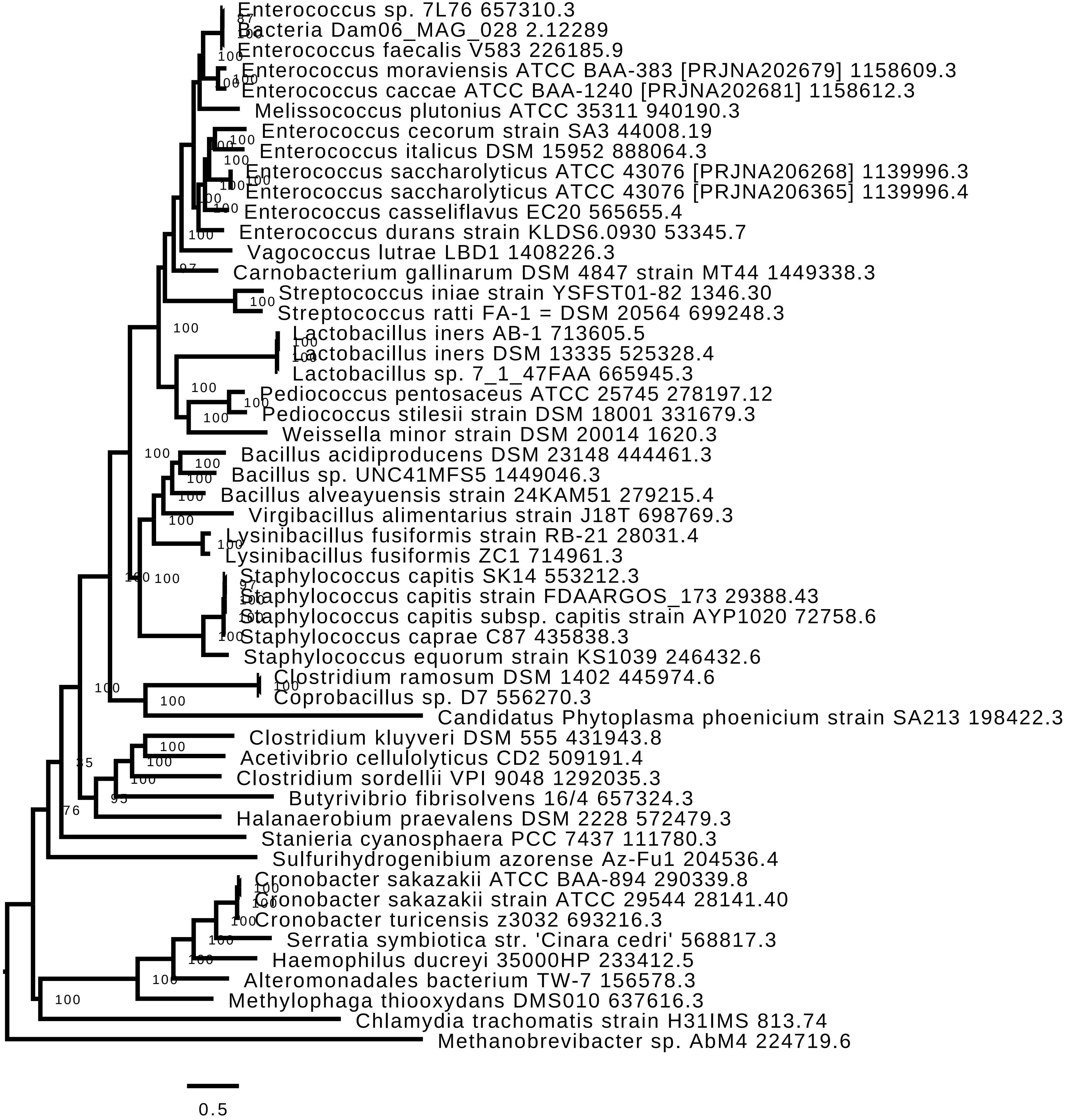
Enterococcus sp. 7L76 657310.3
Bacteria Dam06_MAG_028 2.12289
Enterococcus faecalis V583 226185.9
Enterococcus moraviensis ATCC BAA-383 [PRJNA202679] 1158609.3
Enterococcus caccae ATCC BAA-1240 [PRJNA202681] 1158612.3
Melissococcus plutonius ATCC 35311 940190.3
Enterococcus cecorum strain SA3 44008.19
Enterococcus italicus DSM 15952 888064.3
Enterococcus saccharolyticus ATCC 43076 [PRJNA206268] 1139996.3
Enterococcus saccharolyticus ATCC 43076 [PRJNA206365] 1139996.4
Enterococcus casseliflavus EC20 565655.4
Enterococcus durans strain KLDS6.0930 53345.7
Vagococcus lutrae LBD1 1408226.3
Carnobacterium gallinarum DSM 4847 strain MT44 1449338.3
Streptococcus iniae strain YSFST01-82 1346.30
Streptococcus ratti FA-1 = DSM 20564 699248.3
Lactobacillus iners AB-1 713605.5
Lactobacillus iners DSM 13335 525328.4
Lactobacillus sp. 7_1_47FAA 665945.3
Pediococcus pentosaceus ATCC 25745 278197.12
Pediococcus stilesii strain DSM 18001 331679.3
Weissella minor strain DSM 20014 1620.3
Bacillus acidiproducens DSM 23148 444461.3
Bacillus sp. UNC41MFS5 1449046.3
Bacillus alveayuensis strain 24KAM51 279215.4
Virgibacillus alimentarius strain J18T 698769.3
Lysinibacillus fusiformis strain RB-21 28031.4
Lysinibacillus fusiformis ZC1 714961.3
Staphylococcus capitis SK14 553212.3
Staphylococcus capitis strain FDAARGOS_173 29388.43
Staphylococcus capitis subsp. capitis strain AYP1020 72758.6
Staphylococcus caprae C87 435838.3
Staphylococcus equorum strain KS1039 246432.6
Clostridium ramosum DSM 1402 445974.6
Coprobacillus sp. D7 556270.3
Candidatus Phytoplasma phoenicium strain SA213 198422.3
Clostridium kluyveri DSM 555 431943.8
Acetivibrio cellulolyticus CD2 509191.4
Clostridium sordellii VPI 9048 1292035.3
Butyrivibrio fibrisolvens 16/4 657324.3
Halanaerobium praevalens DSM 2228 572479.3
Stanieria cyanosphaera PCC 7437 111780.3
Sulfurihydrogenibium azorense Az-Fu1 204536.4
Cronobacter sakazakii ATCC BAA-894 290339.8
Cronobacter sakazakii strain ATCC 29544 28141.40
Cronobacter turicensis z3032 693216.3
Serratia symbiotica str. 'Cinara cedri' 568817.3
Haemophilus ducreyi 35000HP 233412.5
Alteromonadales bacterium TW-7 156578.3
Methylophaga thiooxydans DMS010 637616.3
Chlamydia trachomatis strain H31IMS 813.74
Methanobrevibacter sp. AbM4 224719.6
0.5

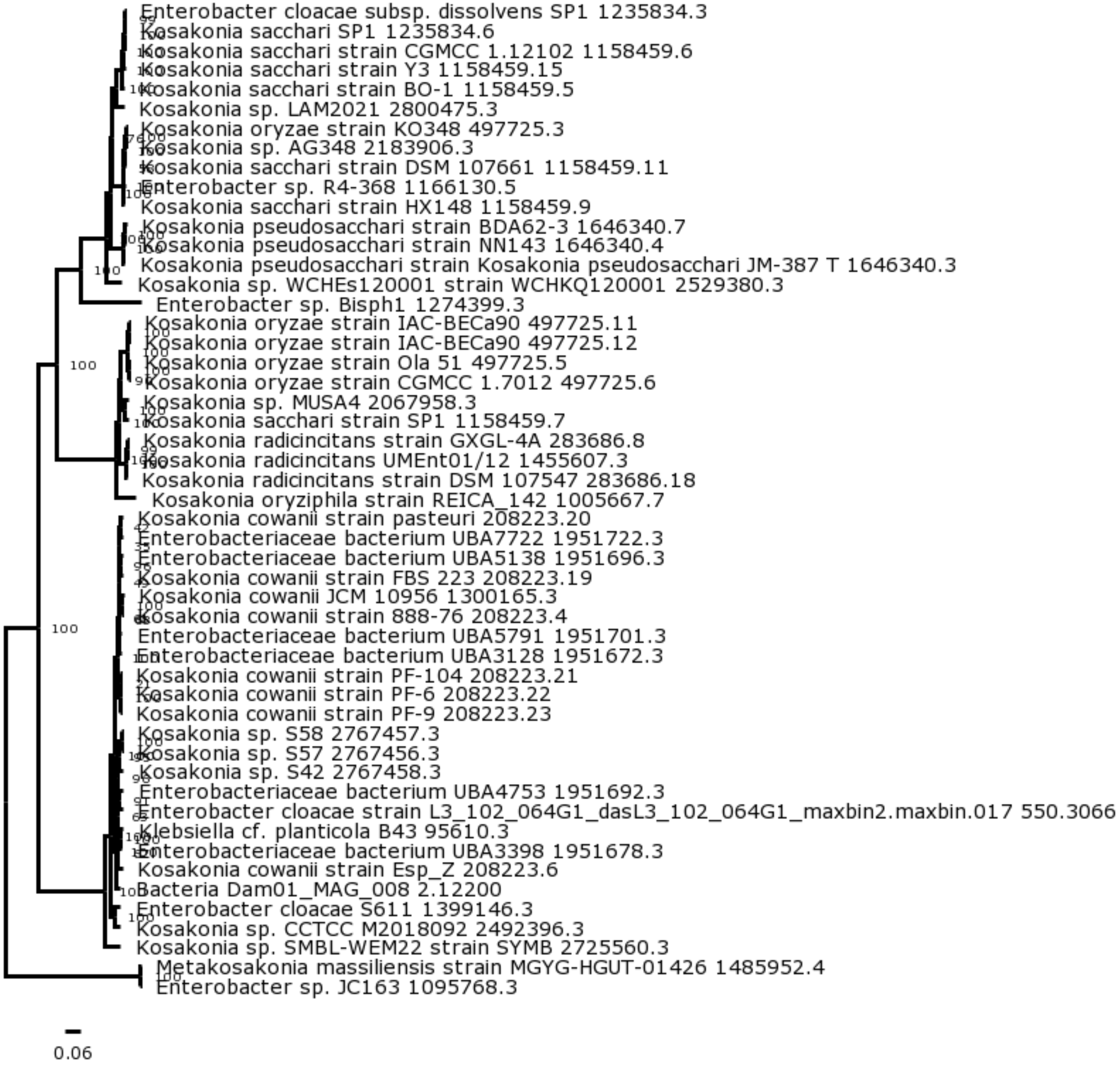
Enterobacter cloacae subsp. dissolvens SP1 1235834.3
Kosakonia sacchari SP1 1235834.6
Kosakonia sacchari strain CGMCC 1.12102 1158459.6
Kosakonia sacchari strain Y3 1158459.15
Kosakonia sacchari strain BO-1 1158459.5
Kosakonia sp. LAM2021 2800475.3
Kosakonia oryzae strain KO348 497725.3
Kosakonia sp. AG348 2183906.3
Kosakonia sacchari strain DSM 107661 1158459.11
Enterobacter sp. R4-368 1166130.5
Kosakonia sacchari strain HX148 1158459.9
Kosakonia pseudosacchari strain BDA62-3 1646340.7
Kosakonia pseudosacchari strain NN143 1646340.4
Kosakonia pseudosacchari strain Kosakonia pseudosacchari JM-387 T 1646340.3
Kosakonia sp. WCHEs120001 strain WCHKQ120001 2529380.3
Enterobacter sp. Bisph1 1274399.3
Kosakonia oryzae strain IAC-BECa90 497725.11
Kosakonia oryzae strain IAC-BECa90 497725.12
Kosakonia oryzae strain Ola 51 497725.5
Kosakonia oryzae strain CGMCC 1.7012 497725.6
Kosakonia sp. MUSA4 2067958.3
Kosakonia sacchari strain SP1 1158459.7
Kosakonia radicincitans strain GXGL-4A 283686.8
Kosakonia radicincitans UMEnt01/12 1455607.3
Kosakonia radicincitans strain DSM 107547 283686.18
Kosakonia oryziphila strain REICA_142 1005667.7
Kosakonia cowanii strain pasteuri 208223.20
Enterobacteriaceae bacterium UBA7722 1951722.3
Enterobacteriaceae bacterium UBA5138 1951696.3
Kosakonia cowanii strain FBS 223 208223.19
Kosakonia cowanii JCM 10956 1300165.3
Kosakonia cowanii strain 888-76 208223.4
Enterobacteriaceae bacterium UBA5791 1951701.3
Enterobacteriaceae bacterium UBA3128 1951672.3
Kosakonia cowanii strain PF-104 208223.21
Kosakonia cowanii strain PF-6 208223.22
Kosakonia cowanii strain PF-9 208223.23
Kosakonia sp. S58 2767457.3
Kosakonia sp. S57 2767456.3
Kosakonia sp. S42 2767458.3
Enterobacteriaceae bacterium UBA4753 1951692.3
Enterobacter cloacae strain L3_102_064G1_dasL3_102_064G1_maxbin2.maxbin.017 550.3066
Klebsiella cf. planticola B43 95610.3
Enterobacteriaceae bacterium UBA3398 1951678.3
Kosakonia cowanii strain Esp_Z 208223.6
Bacteria Dam01_MAG_008 2.12200
Enterobacter cloacae S611 1399146.3
Kosakonia sp. CCTCC M2018092 2492396.3
Kosakonia sp. SMBL-WEM22 strain SYMB 2725560.3
Metakosakonia massiliensis strain MGYG-HGUT-01426 1485952.4
Enterobacter sp. JC163 1095768.3
0.06

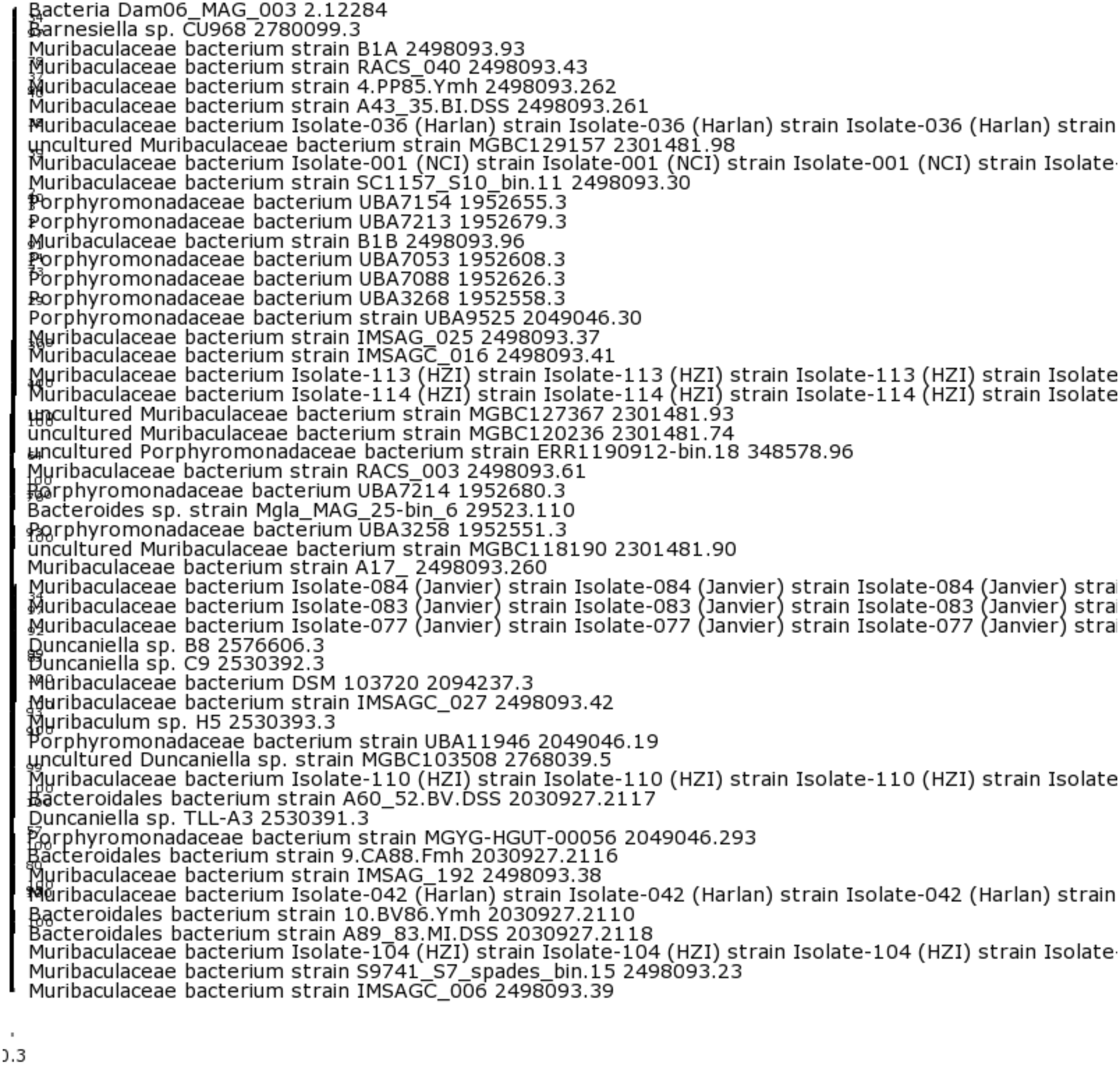
Bacteria Dam06_MAG_003 2.12284
Barnesiella sp. CU968 2780099.3
Muribaculaceae bacterium strain B1A 2498093.93
Muribaculaceae bacterium strain RACS_040 2498093.43
Muribaculaceae bacterium strain 4.PP85.Ymh 2498093.262
Muribaculaceae bacterium strain A43_35.BI.DSS 2498093.261
Muribaculaceae bacterium Isolate-036 (Harlan) strain Isolate-036 (Harlan) strain Isolate-036 (Harlan) strain
uncultured Muribaculaceae bacterium strain MGBC129157 2301481.98
Muribaculaceae bacterium Isolate-001 (NCI) strain Isolate-001 (NCI) strain Isolate-001 (NCI) strain Isolate-
Muribaculaceae bacterium strain SC1157_S10_bin.11 2498093.30
Porphyromonadaceae bacterium UBA7154 1952655.3
Porphyromonadaceae bacterium UBA7213 1952679.3
Muribaculaceae bacterium strain B1B 2498093.96
Porphyromonadaceae bacterium UBA7053 1952608.3
Porphyromonadaceae bacterium UBA7088 1952626.3
Porphyromonadaceae bacterium UBA3268 1952558.3
Porphyromonadaceae bacterium strain UBA9525 2049046.30
Muribaculaceae bacterium strain IMSAG_025 2498093.37
Muribaculaceae bacterium strain IMSAGC_016 2498093.41
Muribaculaceae bacterium Isolate-113 (HZI) strain Isolate-113 (HZI) strain Isolate-113 (HZI) strain Isolate
Muribaculaceae bacterium Isolate-114 (HZI) strain Isolate-114 (HZI) strain Isolate-114 (HZI) strain Isolate
uncultured Muribaculaceae bacterium strain MGBC127367 2301481.93
uncultured Muribaculaceae bacterium strain MGBC120236 2301481.74
uncultured Porphyromonadaceae bacterium strain ERR1190912-bin.18 348578.96
Muribaculaceae bacterium strain RACS_003 2498093.61
Porphyromonadaceae bacterium UBA7214 1952680.3
Bacteroides sp. strain Mgla_MAG_25-bin_6 29523.110
Porphyromonadaceae bacterium UBA3258 1952551.3
uncultured Muribaculaceae bacterium strain MGBC118190 2301481.90
Muribaculaceae bacterium strain A17_ 2498093.260
Muribaculaceae bacterium Isolate-084 (Janvier) strain Isolate-084 (Janvier) strain Isolate-084 (Janvier) strai
Muribaculaceae bacterium Isolate-083 (Janvier) strain Isolate-083 (Janvier) strain Isolate-083 (Janvier) strai
Muribaculaceae bacterium Isolate-077 (Janvier) strain Isolate-077 (Janvier) strain Isolate-077 (Janvier) strai
Duncaniella sp. B8 2576606.3
Duncaniella sp. C9 2530392.3
Muribaculaceae bacterium DSM 103720 2094237.3
Muribaculaceae bacterium strain IMSAGC_027 2498093.42
Muribaculum sp. H5 2530393.3
Porphyromonadaceae bacterium strain UBA11946 2049046.19
uncultured Duncaniella sp. strain MGBC103508 2768039.5
Muribaculaceae bacterium Isolate-110 (HZI) strain Isolate-110 (HZI) strain Isolate-110 (HZI) strain Isolate
Bacteroidales bacterium strain A60_52.BV.DSS 2030927.2117
Duncaniella sp. TLL-A3 2530391.3
Porphyromonadaceae bacterium strain MGYG-HGUT-00056 2049046.293
Bacteroidales bacterium strain 9.CA88.Fmh 2030927.2116
Muribaculaceae bacterium strain IMSAG_192 2498093.38
Muribaculaceae bacterium Isolate-042 (Harlan) strain Isolate-042 (Harlan) strain Isolate-042 (Harlan) strain
Bacteroidales bacterium strain 10.BV86.Ymh 2030927.2110
Bacteroidales bacterium strain A89_83.MI.DSS 2030927.2118
Muribaculaceae bacterium Isolate-104 (HZI) strain Isolate-104 (HZI) strain Isolate-104 (HZI) strain Isolate-
Muribaculaceae bacterium strain S9741_S7_spades_bin.15 2498093.23
Muribaculaceae bacterium strain IMSAGC_006 2498093.39
0.3
