# Supplementary material for: Limitation of amino acid availability by bacterial populations during enhanced colitis in IBD mouse model
Source: mSystems. 2023 Nov 1;8(6):e00703-23. doi: 10.1128/msystems.00703-23 (PMC10746178; doi:10.1128/msystems.00703-23)
Supplement: Figure S4 — Nutrient dependency assay results for each isolate. [file msystems.00703-23-s0004.pdf]

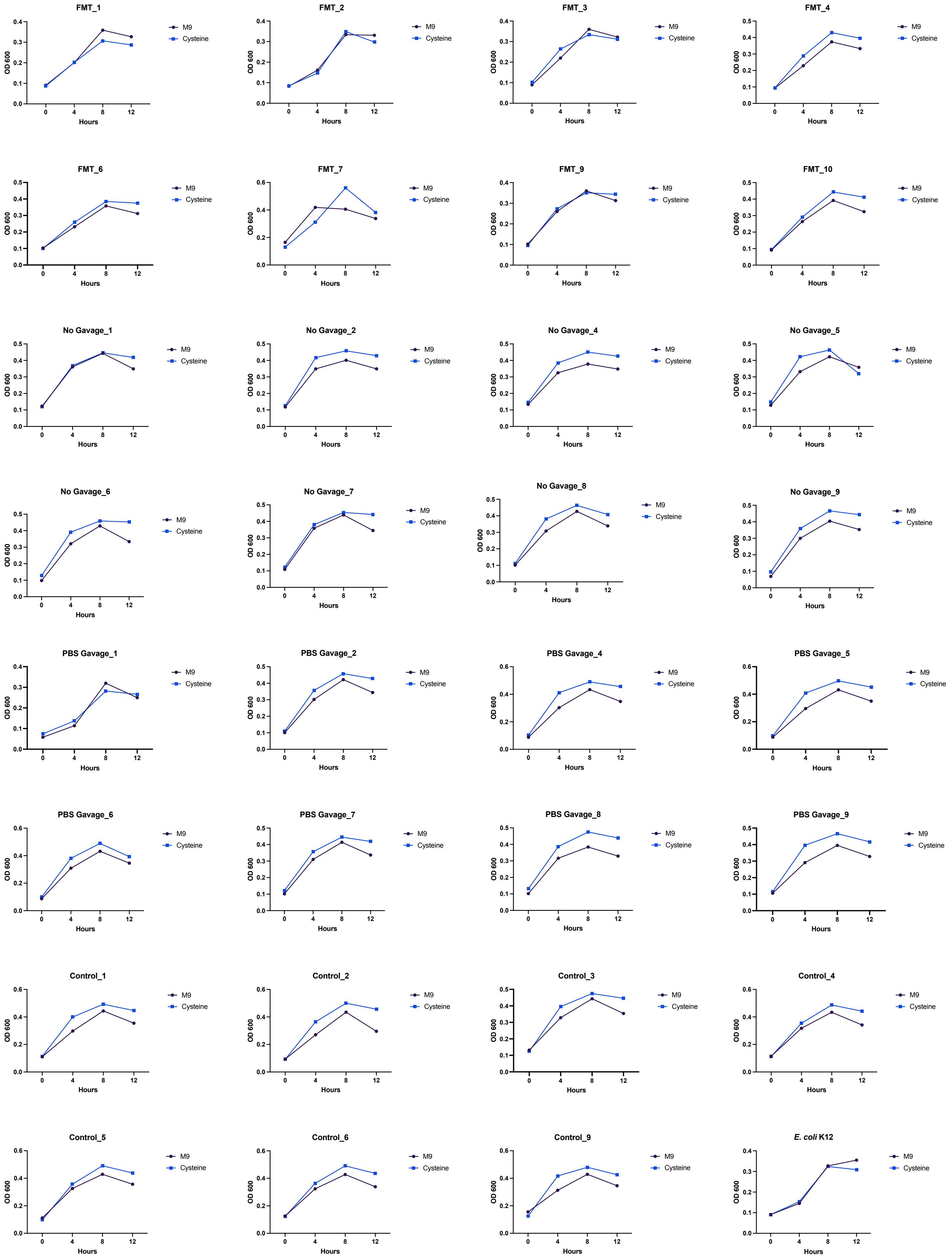

Supplementary Figure S4. Growth curves of each bacterial isolate named by the original mouse treatment group over 12 hours comparing minimal media to media supplemented with 100 uM of L-cysteine.
